# CSCA-YOLOv8: A lightweight network model for evaluating drought resistance in mung bean

**DOI:** 10.1371/journal.pone.0326328

**Published:** 2025-07-31

**Authors:** Dongshan Jiang, Jinyang Liu, Haomiao Zhang, Wenxiang Liang, Ziqiu Luo, Wenlong An, Shicong Li, Xin Chen, Xingxing Yuan, Shangbing Gao

**Affiliations:** 1 Department of Computer and Software Engineering, Huaiyin Institute of Technology, Huaian, China; 2 Jiangsu Academy of Agricultural Sciences, Jiangsu Key Laboratory for Horticultural Crop Genetic Improvement, Institute of Industrial Crops, Nanjing, Jiangsu, China; Gomal University, PAKISTAN

## Abstract

Drought is one of the main factors affecting mung bean production in China. Screening drought-resistant germplasm resources and cultivating drought-resistant varieties are of great significance to the development of the mung bean industry in China. Combined with chlorophyll fluorescence imaging technology, this paper proposes a lightweight mung bean drought resistance identification network model based on YOLOv8, referred to as CSCA-YOLOv8. The model uses StarNet to replace the backbone network of YOLOv8 to reduce the size of the model. The C2f_Star module is introduced in the neck structure instead of the original C2f module. Then, in order to enhance the network’s attention to the key regions in the feature map, the Context Anchor Attention Mechanism (CAA) module is also introduced into the fourth C2f_Star module. Then, a CGBD module is proposed in the neck structure to reconstruct the ordinary convolution to improve the feature extraction ability of the model for small targets. Finally, the SIoU loss function is used to replace CIoU to accelerate the convergence of the model. In the actual data analysis, we used the collected 4808 chlorophyll fluorescence images of the natural mung bean population under drought stress to make the Mungbean Drought Datatset(MDD) and made classification labels for each image according to different drought resistance levels, which were 0, 1, 2, 3, 4 and 5. We also verified the excellent performance and generalization performance of the model using the collected MDD dataset. The final experimental results show that compared with the YOLOv8s baseline model, the number of parameters of our proposed algorithm is reduced by 24%, the floating point number is reduced by 35%, and the accuracy is improved by 2.52%, which supports the deployment on embedded edge devices with limited computing power. Therefore, our proposed algorithm has great potential in the field of drought resistance identification and germplasm selection of mung bean.

## Introduction

Mung bean is one of the most important grain leguminous crops in Asia, and also an important small grain crop in China [1]. However, the urban heat island and dry island effects work together to accelerate surface water evaporation and lead to a dry climate [2–4]. Under this background, drought stress has increasingly become a severe environmental challenge, even affecting the growth of drought-resistant plants [5]. Affected by drought stress, mung bean crop cells show dehydration shrinkage and cell wall hardening [6], which seriously leads to a significant decline in mung bean yield [7].Therefore, in order to effectively deal with drought disasters, it is of great strategic significance to develop efficient and accurate identification methods for drought resistance of mungbean and screen the germplasm resources of drought tolerant mungbean to promote the utilization of dry land and industrial development.

At present, indicators for assessing drought tolerance of plants are mainly divided into physiological characteristics and morphology [8–10]. Although high detection accuracy can be obtained by using physiological characteristic indicators, its operation is complex and will cause different degrees of damage to crops. However, morph-based detection methods are widely used to identify crop stress degree due to their advantages of non-destructive and fast speed. Chlorophyll fluorescence imaging technology can reflect the photosynthetic information of the leaves, such as the absorption and conversion of light energy, the transmission and distribution of energy, and the state of reaction centers. The chlorophyll fluorescence parameters can be used to identify the stress characteristics before the naked eye can see the symptoms, which has been widely used in the monitoring and early warning of various plant stress states [11–12]. Wang et al. investigated soybean seedlings to analyze changes in chlorophyll fluorescence parameters under both mild and severe drought stress. Their results demonstrated that the actual photochemical quantum yield (ΦPSII) exhibited the highest correlation with drought intensity. Based on this parameter, they successfully identified drought-resistant cultivars through screening [13]. Liang et al. compared the drought resistance of different varieties of alfalfa germplasm at the seedling stage through chlorophyll fluorescence parameters, and screened out 14 drought-resistant and high-photoefficiency germplasms from 109 alfalfa samples [14]. These studies indicate that chlorophyll fluorescence imaging is an accurate and reliable method to evaluate the photosynthesis and drought resistance of mung bean using chlorophyll fluorescence parameters. However, most of the chlorophyll fluorescence imaging studies for the evaluation of plant drought tolerance use low-throughput algorithms, and these algorithms cannot automatically extract features in the images, which poses great challenges for the practical application of drought stress detection equipment in production.

In recent years, the rapid development of computer vision and deep learning technologies has brought new opportunities to the field of agriculture. Chandel et al. used AlexNet, GoogLeNet and InceptionV3 models to test the drought stress situation of maize, okole and soybean, and their study showed that GoogLeNet showed superior performance than other models in identifying drought stress of these plants [15]. Xia et al. developed two CNN models, one using six characteristic bands and the other using full spectral bands, for identifying the water stress status of tomato plants [16]. Wang et al. collected multiple parameters of poplar seedlings, such as plant height, ground diameter, and leaf stalk Angle, and used the proposed ResNet18-LSTM and ResNet18-CBAM-LSTM models to realize the multi-output classification of poplar seedling varieties and drought stress levels. The experimental results show that the ResNet18-CBAM-LSTM model has the best performance in the classification of variety screening and drought stress level [17]. Although GoogLeNet, ResNet18-CBAM-LSTM and other methods have achieved some results in drought stress recognition, their processing speed and target detection accuracy are relatively backward. Subsequently, the one-stage detection algorithm You Only Look Once(YOLO) gained popularity in agriculture in a relatively short period of time due to its state-of-the-art performance in terms of accuracy, speed, and network scale [18]. At present, it has been successfully used in field crops such as wheat [19], apple [20], citrus [21], corn [22], grape [23]. The core idea of YOLO is to transform the object detection task into a regression problem, and use a single neural network to simultaneously localize and classify the object, so as to achieve real-time and efficient object detection. However, YOLO algorithm also has some shortcomings, such as the effect is often not ideal when dealing with small targets, and with the increase of model size and running time, the model is difficult to deploy to actual embedded devices.

To address the problems of low recognition rate and large model volume in traditional drought resistance identification methods that are difficult to deploy on hardware, this paper proposes a lightweight drought resistance identification method for mung beans based on chlorophyll fluorescence imaging technology and YOLOv8. This method is an improvement of YOLOv8s network model, and uses lightweight design to achieve an effective balance between model accuracy and processing speed. It can be an effective tool for accurately detecting the drought resistance characteristics of mung beans in real complex field environments. The main contributions of this paper are as follows:

(1) The StarNet network is introduced into the backbone network, which is a four-stage hierarchical architecture. The convolutional layer is used to reduce the resolution of the feature map, and multiple Star_Block modules are reused for feature extraction.
(2) In the neck part, the proposed C2f_Star module is used to replace the original C2f modules in the first, second and third positions to further reduce the size of the model. At the same time, the context anchor attention mechanism CAA is introduced. The CAA attention mechanism uses global average pooling and bar convolution to enhance the features of the central region, so that the model can capture key information more accurately, thereby improving the detection accuracy.
(3) The feature fusion module CGBD module is constructed to replace the ordinary convolution, which not only extracts and fuses the local features and context features, but also retains and enhances the information in the feature map during the downsampling process.
(4) A MDD dataset for the identification of drought resistance of mung bean was constructed. As far as I know, this dataset is the first image dataset for the identification of drought resistance of crops. The dataset and source code are available on Github.

## Materials and methods

### Material source and experimental design

The natural population materials of mungbean used in the experiment were from the Legumbean Laboratory of the Institute of Cash Crops, Jiangsu Academy of Agricultural Sciences. The breeding work was carried out in the intelligent greenhouse of the Institute of Cash Crops of Jiangsu Academy of Agricultural Sciences from July to August 2024, and 235 mung bean germplasm resources were treated with drought stress. To ensure the healthy growth of mung bean seedlings, the intelligent greenhouse needs to be sterilized before the experiment. The specific experimental steps are as follows: (1) Select 20 undamaged, uniformly sized and plump mung bean seeds from each variety. Disinfect the mung bean seeds with 20% hydrogen peroxide solution and then rinse them clean with distilled water. (2) Sow them in plastic POTS of 10 × 10 cm, add an appropriate amount of water every day, and record the germination situation every day. (3) Polyethylene glycol solution (PEG6000) is a reagent often used to simulate water stress in vitro. Due to its high solubility in water, it is widely applied in the study of the responses and adaptation mechanisms of plants under water stress conditions [24–26]. After the seeds of mung bean grew new leaves on the fifth day of hydroponics, this study used the experimental method of PEG-6000 to simulate drought stress, and added 20% PEG6000 solution in 40 ml per group to each treatment group, and observed the growth of mung bean seedlings [27]. Similarly, 40 ml of water was added to each CK group to ensure the normal growth of the control group. (4) On the fifth and tenth day after stress treatment, three plants of each mung bean variety were taken from the treatment and the control, respectively, and placed on the tray of the chlorophyll fluorescence imaging system (FluorCam), and then FluorCam7 software was used for image acquisition and data analysis.

### Image acquisition

**Fig 1:**
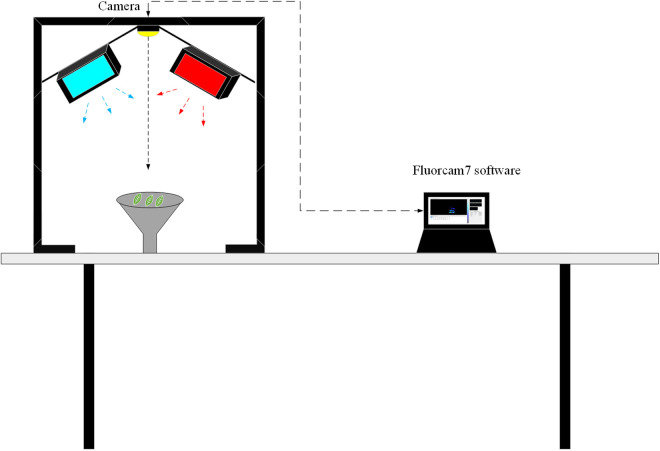
Schematic diagram of the experimental collection device.

The FluorCam chlorophyll fluorescence imaging system is a highly flexible and widely applied plant physiological and ecological research instrument [28]. The schematic diagram of the experimental acquisition device are shown in Fig 1. It can be seen that this system adopts a modular structure and is mainly composed of four LEDs light source plates, CCD lenses, brackets, control units and FluorCam imaging analysis software. The specific image acquisition steps are as follows.

(1) Experiment preparation: Before the experiment, the hardware equipment of the chlorophyll fluorescence imaging system should be proofread according to the instructions to ensure the accuracy of the fluorescence signal. Place the sample in a completely dark environment for 15–20 minutes to ensure that the sample reaches a fully dark adaptation state. Then place the mung bean samples on the sample stage, ensuring that the leaves are flat and fully cover the imaging area.
(2) Experimental parameter setting: Start FluorCam software and select Fv/Fm mode in the Protocol interface. Then, turn on Flashes and set Shutter to 2 and Sensitivity to 60.
(3) Data and image acquisition: After all parameters are set, click the “Start” button in the software, and the system will automatically perform fluorescence excitation and data acquisition. Observe the fluorescence images in real time in the software to ensure that the sample position and signal strength are appropriate.After the collection is completed, save the fluorescence images and the original data.
(4) Data analysis: Import the saved data into the FluorCam analysis software, select the analysis module as needed, and generate results and images.

### Image preprocessing

To avoid the interference of irrelevant information in the images, FluorCam 7.0 software was used to select the region of interest and perform background segmentation processing on each collected fluorescence image. In addition, we set a threshold interval of 0.2–0.85 for the chlorophyll fluorescence parameter Fv/Fm obtained by software analysis, so that all pixels of the image fluctuate within this interval. We also use Labelimg to manually label the targets in each fluorescence image according to different drought resistance levels and save the label processed data into the original data set [29]. In total, the original dataset contained 1202 fluorescence images with at least two labels per image.

**Fig 2:**
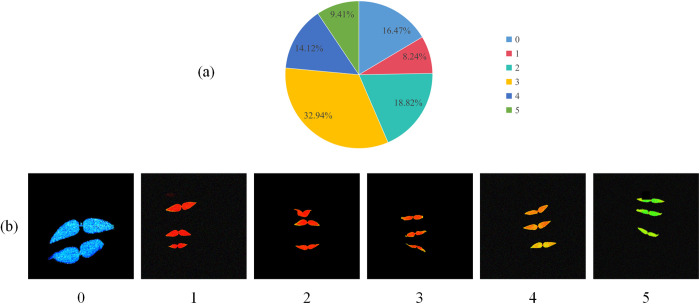
(a) Proportion of the number of labels with different drought resistance levels in the MDD dataset. (b) Fluorescence images of different drought resistance levels.

In order to solve the problem of insufficient data samples or unbalanced data, we used data enhancement methods such as rotation, translation, changing brightness, adding noise, cutout, flipping, and mosaic to expand the labeled original data, and obtained the data augmented dataset [30–31]. Then we split the MDD into training, validation, and test sets with a ratio of 8:1:1. In the divided MDD dataset, there are 3846 images in the training set, 481 in the validation set, and 481 in the test set. Fig 2(a) shows the proportion of the number of labels of different drought resistance levels in the dataset. From the figure, we can see that the labels of category 3 account for 32.94% of the dataset, while the number of labels of category 1 is the least.

### Criteria for drought resistance identification

Under drought stress conditions, mung beans with stronger drought resistance are usually less affected by drought stress and can demonstrate better adaptability and tolerance. The maximum light quantum efficiency Fv/Fm value can reflect the response degree of crops to drought stress, and the lower Fv/Fm value represents the weaker resistance of crops to drought stress [32]. Therefore, the drought resistance of mung bean can be identified by using the chlorophyll image information of the early seedling, so that high-quality and drought-resistant germplasm resources can be selected for breeding. In this study, the drought resistance characteristics of mung beans were evaluated based on the maximum photochemical efficiency Fv/Fm value. The specific drought resistance classification criteria are shown in Table 1. Fig 2(b) shows some chlorophyll fluorescence images of different drought resistance levels.

**Table 1:** Classification standards for drought resistance levels in mungbeans.

Levels	Fv/Fm	Drought resistance	Characteristics of leaf
0	CK group		Leaves grow normally without drought stress.
1	>0.83	Highly Resistance (HR)	Leaves extend naturally, emerald or dark green in color.
2	0.82 ~ 0.83	Resistance (R)	Leaf margin slightly wrinkles.
3	0.79 ~ 0.81	Moderate Resistance (MR)	Leaf margin curls, leaf surface wrinkles.
4	0.70 ~ 0.78	Sensitive (S)	Leaf curls into tube shape, leaf large area dry, yellow.
5	<0.70	Highly Sensitive (HS)	The leaves lose water and the plants die.

### YOLOv8 model

**Fig 3:**
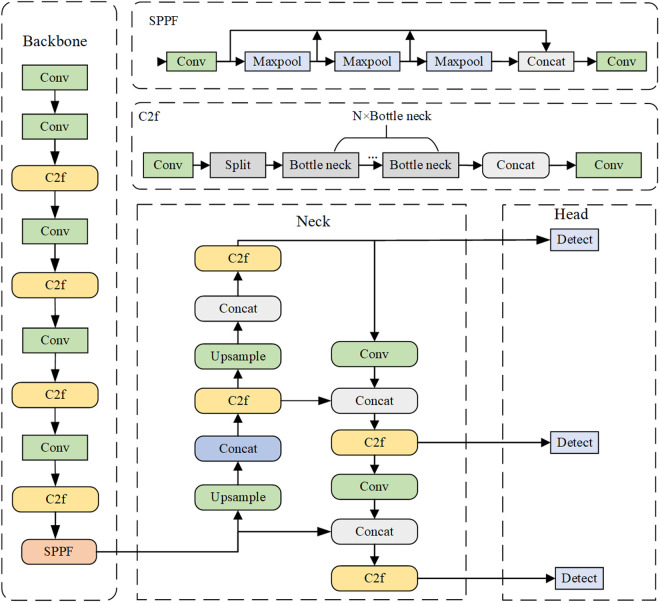
YOLOv8 network structure.

The YOLOv8 algorithm can split the image into cells and predict the class and location of the object in each cell [33]. The overall architecture of YOLOv8 is illustrated in Fig 3. It is mainly composed of three parts: Backbone, Neck and Head. Among them, the Backbone part is mainly used to extract important features from the input image, and then a series of convolution and C2f modules are utilized to extract the deep features in the image. The Backbone part also uses residual connections and bottleneck structure to reduce the size of the network and improve the performance of the model. In the Neck part, the feature pyramid network (FPN) is introduced to enable the model to detect objects at different scales, which improves the accuracy and robustness of the model. The Head part generates the final prediction result. The detection block mainly contains two branches, the first branch for bounding box prediction and the second branch for class prediction. Both of these two branches contain two 3 × 3 convolution and one 1 × 1 convolution, which are used to calculate the bounding box loss and the category loss. According to the model size and depth, the YOLOv8 models mainly include five types: YOLOv8n, YOLOv8s, YOLOv8m, YOLOv8l, and YOLOv8x. With the increase of the depth of the model, the model can obtain richer feature information in the image, thereby increasing the detection accuracy of the model. However, with the increase in model volume and running time, it is difficult to deploy the model to actual embedded devices. Therefore, in order to solve the above problems, this paper selects the YOLOv8s model with smaller model size for lightweight improvement according to the phenotypic stress characteristics of mung bean with different drought resistance levels, so that it can be deployed on embedded devices with small computing power.

### The proposed method

#### StarNet network and Star_Block.

**Fig 4:**
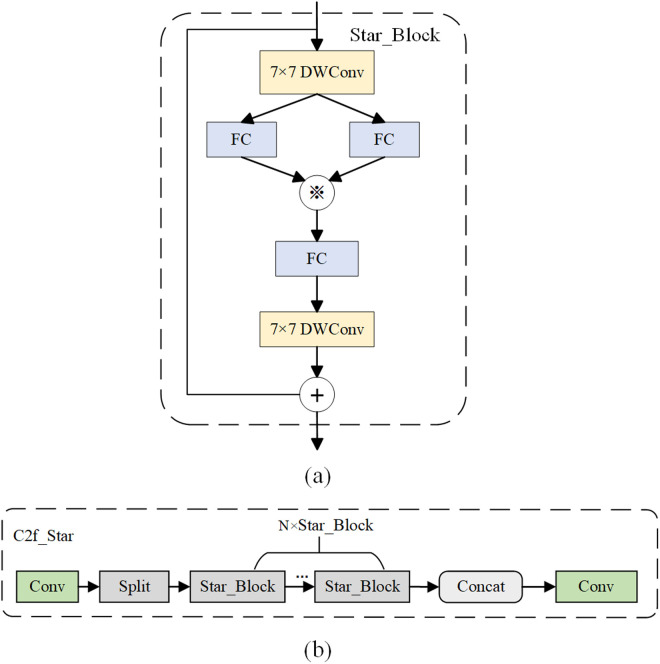
(a) Star_block module. (b) C2f_star module.

With the increase of datasets and application requirements, traditional object detection networks may fail to meet the demands in terms of accuracy and speed. Ma et al. proposed a method called star operation, which fuses different subspace features by element-wise multiplication [34]. In single-layer convolutional neural networks, the star operation is typically written as $\left({W_{1}}^{T}X+B_{1})*({W_{2}}^{T}X+B_{2}\right)$ to denote the fusion of two linear transformations through element-wise multiplication, which has been successfully applied in several domains such as Natural Language Processing and Computer Vision. In order to achieve an effective balance between computational complexity and performance, Ma et al. also proposed a lightweight network called StarNet [34]. The structure of StarNet is a four-stage hierarchical architecture. The convolutional layer is used to reduce the resolution of the feature map, and multiple Star_Block modules are reused for feature extraction. The structure of the Star_Block module is shown in Fig 4(a). In each Star_Block module, a star operation is employed to merge features from two distinct branches. Based on this, this paper replaces the backbone part of YOLOv8s with StarNet to fuse diffFerent subspace features, so as to improve the spatial feature extraction ability of the model. In addition, this paper fuses the C2f module of the neck with Star_Block to form a new C2f_Star module, which aims to achieve model compression and reduce computational requirements. Fig 4(b) shows the schematic structure of the C2f_Star module.

#### Context Anchor Attention mechanism(CAA).

**Fig 5:**
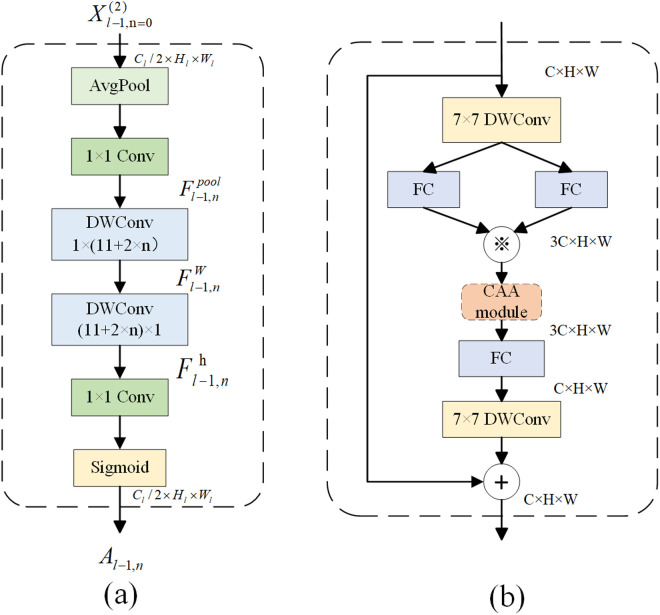
(a) CAA module. (b) Star_Block_CAA module.


Traditional convolutional neural network can extract local feature information in images by convolution operation. However, since the model always allocates the same weight size when extracting image features, the model cannot focus on the key information, resulting in a waste of resource allocation and the loss of a lot of useful information. The attention mechanism is an important technique in the field of deep learning. It enables the model to focus on the important parts of the input feature map, thereby improving the feature expression ability of the model [35]. As a novel attention mechanism, the CAA module enhances contextual information by fixing anchors, which can capture dependencies in images more effectively [36]. As shown in Fig 5(a), the CAA module is mainly composed of an average pooling layer, a common convolutional layer, a depth-wise separable strip convolution, and a Sigmoid activation function. The specific calculation formula is shown in Eqs 1–4. In Eq (1), the global average pooling layer and a 1 × 1 convolutional layer are used to maximize the acquisition of feature information in local regions of the fluorescence image while reducing the dimension and computational burden. In order to capture the remote context information, the CAA module adopts two depth-separable strip convolutions instead of one depth-separable convolution. This design can significantly increase the receptive field while ensuring lightweight. Finally, the CAA module generates an attention weight to further enhance the output of Star_Block. Based on the concept of Star_Block module and attention mechanism, this paper further optimizes the structure of Star_Block and proposes the Star_Block_CAA module. The structure diagram of Star_Block_CAA module is shown in Fig 5(b).

$$F_{l-1,n}^{pool}=Conv_{1\times 1}(P_{avg}(X_{l-1,n}^{\left(2\right)})),n=0,...,N_{l-1},$$ (1)

where,$P_{avg}$ represents the average pooling operation,$X_{l-1,n}^{\left(2\right)}$ represents the input feature vector, when n = 0,$X_{l-1,n}^{\left(2\right)}=X_{l-1}^{\left(2\right)}$.


$$F_{l-1,n}^{w}=DWConv_{1\times k_{b}}(F_{l-1,n}^{pool}),$$ (2)

$$F_{l-1,n}^{h}=DWConv_{k_{b}\times 1}(F_{l-1,n}^{w}).$$ (3)

$$A_{l-1,n}=Sigmoid(Conv_{1\times 1}(F_{l-1,n}^{h})),$$ (4)

where, the Sigmoid function is utilized to ensure that the feature map stays within the limits of 0–1.

#### CGBD module.

**Fig 6:**
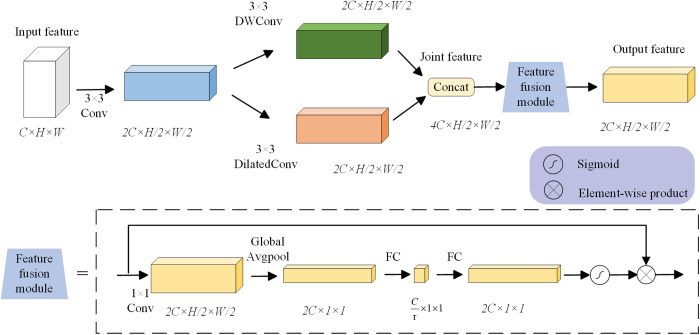
The working principle of the CGBD module.

In order to improve the recognition ability of the model for different drought resistance levels, this paper designs a feature fusion module, called ContextGuidedBlock_Down (CGBD) module. Fig 6 explains the working principle of the CGBD module. Specifically, a 3 × 3 convolution kernel is used to perform convolution operations on the input feature vectors. The size of the feature map is reduced by half, and the number of channels is doubled. The 3 × 3 depthwise separable convolution reduces the number of parameters needed for convolution calculation by splitting the correlation between spatial dimension and channel dimension. The dilated convolution with dilation rate 2 is not only used to extract image stress features with a larger receptive field, but also can improve the robustness of the model. The feature fusion module further refines the key features in the feature map to obtain joint features. The feature fusion module is made up of a common convolution layer, a global average pooling layer, two Fully-connected Layer and a Sigmoid activation function. To sum up, in this paper, the CGBD module is introduced in the neck module to replace the ordinary convolution in the neck module. This not only extracts and fuses local features and context features, but also retains and enhances the information in the feature map during the downsampling process.

#### SIoU loss function.

In the YOLO object detection framework, the design of the loss function is crucial for the effectiveness of the model performance [37]. Traditional object detection loss functions (such as CIoU and GIoU) mainly rely on the distance between the predicted box and the real box, overlap area and aspect ratio, etc., but lack of consideration of the vector Angle between the real box and the predicted box, which makes the model converge slowly and even leads to the model not learning useful features and reduces the performance of the model. In this paper, we introduce an advanced loss function (SIoU) for bounding box regression [38].On the basis of traditional IoU, it considers the scale and Angle information of the boundary box to improve the convergence speed and prediction accuracy of the model. The SIoU loss function is made up of four primary components: angle loss (Lα), distance loss (Ld), shape loss (Ls), and IoU loss. The definition of this overall loss function is as follows.


$$L_{SIoU}=1-IoU+\frac{L_{\alpha }+L_{d}}{2}$$ (5)

where, the formula for the IoU loss function is as follows.


$$IoU=\frac{\left|B\cap B^{GT}\right|}{\left|B\cup B^{GT}\right|}$$ (6)

The formula for the Angle loss is as follows.


$$\left\{ \begin{array}{c} \varphi =\arcsin(\frac{{\text{r}}_{h}}{\delta })-\frac{\pi }{4}\\ L_{\alpha }=1-2*\text{sin}\varphi =\cos(2*\text{sin}\varphi )\\ \frac{{\text{r}}_{h}}{\delta }=\sin(\alpha )\\ \delta =\sqrt{{\left(X_{{\text{r}}_{\text{x}}}^{gt}-X_{{\text{r}}_{\text{x}}}\right)}^{2}+{\left(X_{{\text{r}}_{y}}^{gt}-X_{{\text{r}}_{y}}\right)}^{2}}\\ {\text{r}}_{h}=\max(X_{{\text{r}}_{y}}^{gt},X_{{\text{r}}_{y}})-\min(X_{{\text{r}}_{\text{x}}}^{gt},X_{{\text{r}}_{\text{x}}}) \end{array}\right.\;$$ (7)

where, sin(α) denotes opposite over hypotenuse in a right triangle. δ refers to the distance from the center of the actual box to that of the predicted box.${\;r}_{h}$ represents the vertical difference in position between the center points of the actual and predicted boxes. $X_{rx}^{gt},X_{\begin{array}{c} ry \end{array}}^{gt}$ is the center coordinate of the actual box, and $X_{rx}{,X}_{ry}$ is the center coordinate of the predicted box.

The formula for distance loss is as follows.


$$\left\{ \begin{array}{c} L_{\text{d}}=\sum_{\text{t}=\text{x},y}\left(1-\text{exp(}-\eta \mu \text{t)}\right)=2-\exp(-\eta \mu_{\text{x}})-\exp(-\eta \mu_{\text{y}})\\ \mu_{\text{x}}={\left(\frac{X_{{\text{r}}_{x}}^{gt}-X_{{\text{r}}_{x}}}{{\text{r}}_{w}}\right)}^{2},\mu_{\text{y}}={\left(\frac{X_{{\text{r}}_{\text{y}}}^{gt}-X_{{\text{r}}_{\text{y}}}}{{\text{r}}_{h}}\right)}^{2}\eta =2-L_{\alpha } \end{array}\right.\;$$ (8)

where, $r_{w},r_{h}$ are the width and height of the minimum outer matrix of the actual and predicted boxes.

The shape loss formula is defined as follows:


$$\left\{ \begin{array}{c} L_{s}=\sum_{t=w,h}{\left(1-exp(-w_{t})\right)}^{\theta }=(1-exp(-w_{w}){)}^{\theta }+(1-exp(-w_{h}){)}^{\theta }\\ w_{w}=\frac{\left|w-w^{gt}\right|}{\max(w,w^{gt})},w_{h}=\frac{\left|h-h^{gt}\right|}{\max(h,h^{gt})} \end{array}\right.$$ (9)

where, ${w,h,w}^{gt},h^{gt}$ are the width and height of the predicted and actual boxes, regulating the extent of emphasis placed on the shape condition loss. To prevent excessive concentration on the shape loss and to maintain the movement of the prediction box, the parameter θ range from 2 to 6.

### CSCA-YOLOv8 network model

**Fig 7:**
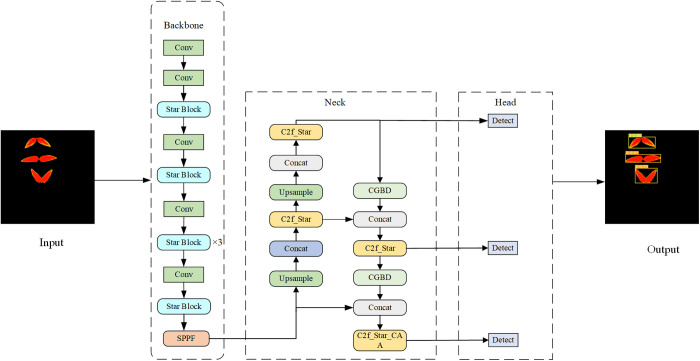
CSCA-YOLOv8 network structure.

This paper makes improvements on the original YOLOv8s network model. The original backbone network is replaced by the lightweight StarNet network. This not only significantly reduces the computational complexity of the model, but also greatly improves the representation ability and performance of the model. In neck part, using C2f_Star module to replace the position 1, 2, 3 on C2f module, to further reduce model size. The C2f_Star_CAA module is used to replace the fourth C2f module to enhance the sensitivity of the model to the target location. The feature fusion module CGBD module is constructed to replace the ordinary convolution and improve the expression ability of the model. The SIoU loss function is used to replace the CIoU loss function, which improves the convergence speed and prediction accuracy of the model. The improved method, known as CSCA-YOLOv8, is depicted in Fig 7.

## Results and discussions

### Experimental platform and parameters

In order to ensure the authenticity and effectiveness of the YOLOv8 model algorithm, all experiments in this study are based on the deep learning framework pytorch1.12.1 and run in the same server. The server experiment environment is Intel Xeon Silver 4210R CPU, Quadro RTX 4000 GPU, 11.3.1 CUDA, 3.6.15 python version, and 8G DDR4 RAM. In this study, the batch size is set to 16 and the number of epochs is 200 for all experiments. We also adopt SGD as an optimizer for updating the model parameters in real time. The specific hyperparameter settings are shown in Table 2.

**Table 2:** Hyperparameters settings.

Hyperparameters	Value
lr0(initial learning rate)	0.01
lrf(final learning rate)	0.01
momentum	0.937
warmup_epochs	3.0
warmup_momentum	0.8
weight_decay	0.0005
mosaic	1.0
fliplr(image flip left-right)	0.5

### Evaluation indicators

In order to verify the effectiveness and credibility of CSCA-YOLOv8 algorithm, In this paper, the network depth (Layers), the number of parameters (parameters), computational complexity (FLOPS), Precision(P), Recall(R), Average Precision(AP) and mAP@0.5 are used to evaluate the performance of the model [39–41]. The symbolic meanings involved in each evaluation indicator are shown in Table 3. P is mainly used to evaluate the accuracy of the model prediction. It represents the fraction of all samples predicted to be positive that are actually positive. R is the fraction of all examples that are actually positive that are correctly predicted as positive by the model, and it measures the model’s ability to find the positive class. AP measures the performance of the model in each category by calculating the area of the P-R curve. mAP is used to comprehensively consider the performance of the model on different categories, and its value is between 0–1.The higher the value, the better the model performance in target detection in different drought resistance classes of mung bean. The calculation formula of the specific evaluation index is defined as follows.

**Table 3:** The meanings of the symbols in evaluation indicator.

Symbols	Meanings
T_P_	T_P_ stands for the quantity of positive samples that are accurately recognized.
F_P_	F_P_ indicates the quantity of negative samples that have been incorrectly identified.
F_N_	FN refers to the total of positive samples that are not detected.
IoU	The IoU metric reflects the alignment of the predicted bounding box to the actual one.
classes	class represents the number of categories for object detection by the model
Frames	Frames describes a single image in a video or animation


$$P=\frac{T_{P}}{T_{P}+F_{P}}$$ (10)

$$R=\frac{T_{P}}{T_{P}+F_{N}}$$ (11)

$$AP=\int_{0}^{1}PR\text{dr}$$ (12)

$$\text{m}AP=\frac{1}{\text{classes}}\sum_{\text{i}=0}^{\text{class}}AP_{\text{i}}$$ (13)

Floating-point Operations Per Second(FLOPs) is the number of floating-point operations per second that can be performed. It can represent the computational complexity of the model [42]. The model with more complex network structure has higher FLOPs value. Frames Per Second (FPS) is a standard metric to measure the performance of graphics rendering [43]. The higher the FPS, the faster the model is processed. The formula of the FPS is defined as follows.


$$FPS=\frac{Frames}{Time}$$ (14)

### Model performance analysis

To better compare the detection performance of CSCA-YOLOv8 and YOLOv8 on the self-built MDD dataset, we conducted a series of experiments and used metrics such as P, R, mAP@0.5, Layers, Parameters, FLOPs and FPS to evaluate the performance of the model. The comparative detection results are shown in Table 4. It can be seen from the data in Table 4 that compared with the YOLOv8n and YOLOv8s models, the average detection accuracy of the CSCA-YOLOv8 model is improved by 4.37% and 2.52%, respectively. In terms of Parameters and FLOPs, YOLOv8n has the smallest number of parameters and computational complexity, while its mAP@0.5 and FPS are at the lowest level. This shows that although YOLOv8n is simple to calculate, its detection accuracy and speed are not ideal. Compared with the original YOLOv8s, the CSCA-YOLOv8 model is reduced by 24% and 35%, respectively. And its P and mAP@0.5 values reach the maximum among the three. This shows that the CSCA-YOLOv8 model can not only ensure the accuracy of detection, but also take into account the computational efficiency to a certain extent, showing good comprehensive performance in the recognition task of mung bean drought resistance.

**Table 4:** Comparison results of YOLOv8 and CSCA-YOLOv8 on the MDD dataset.

Model	P (%)	R (%)	mAP@0.5 (%)	Layers	Parameter	FLOPs (G)	FPS
YOLOv8n	86.68	89.70	92.48	225	**3157200**	**8.9**	93.3
YOLOv8s	88.53	90.72	93.62	225	11166560	28.8	132.3
Ours	**91.05**	**92.17**	**94.75**	**280**	8501416	18.6	**134.5**

**Fig 8:**
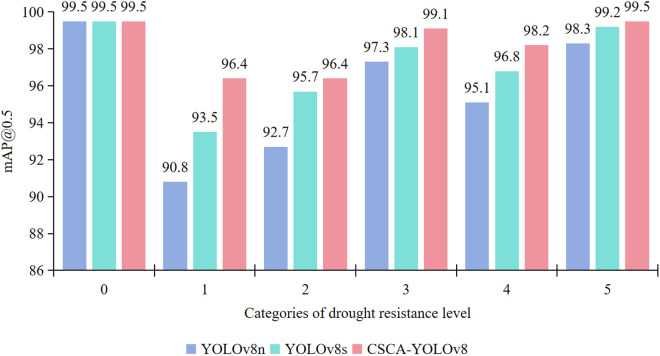
Comparison detection results of the three models on different drought resistance level categories.

Fig 8 shows the mAP@0.5 detection results of YOLOv8n, YOLOv8s, and CSCA-YOLOv8 models on different drought resistance level categories. For the drought resistance level category 0, the mAP@0.5 of the three models all reached 99.5%, indicating that in this category, the three models can achieve extremely high accuracy detection. In category 1, the mAP@0.5 of the CSCA-YOLOv8 model is 96.4%, which is significantly higher than that of YOLOv8n (90.8%) and YOLOv8s (93.5%). This shows that the CSCA-YOLOv8 model can identify the target more accurately and reduce false detection and missed detection when detecting this drought resistance level category. In category 2, the mAP@0.5 of CSCA-YOLOv8 model is also higher than that of YOLOv8n and YOLOv8s, indicating that it can more accurately capture the stress characteristics of this drought-resistant grade crop in this category detection. For categories 3, 4 and 5, the mAP@0.5 of the CSCA-YOLOv8 model is higher than that of YOLOv8n and YOLOv8s, which indicates that the CSCA-YOLOv8 model can better locate and identify the target object when dealing with the target of this drought resistance level. Overall, the CSCA-YOLOv8 model ‘s mAP@0.5 value is higher than that of YOLOv8n and YOLOv8s in all five drought resistance class categories except class 0, which fully demonstrates that the method proposed in this paper significantly improves the detection accuracy for different drought resistance class categories.

**Fig 9:**
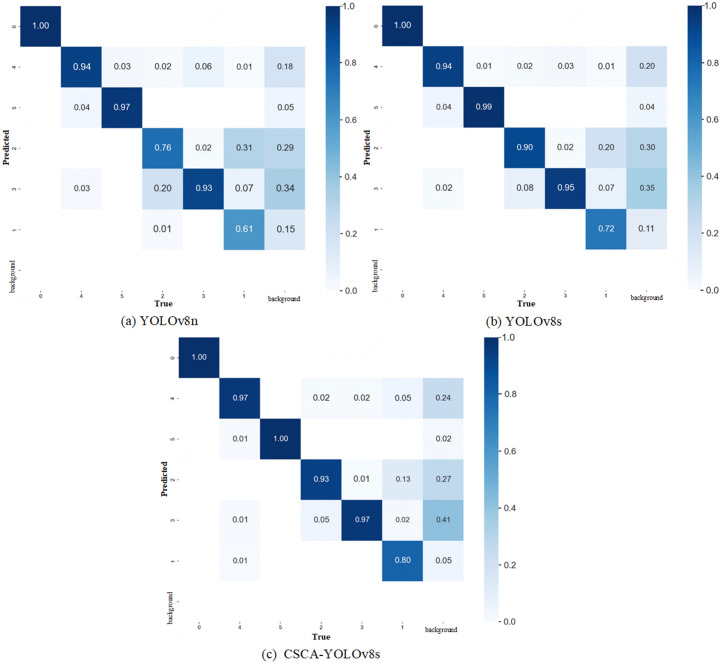
Confusion matrix of YOLOv8n,YOLOv8s and CSCA-YOLOv8 on the MDD dataset.

**Fig 10:**
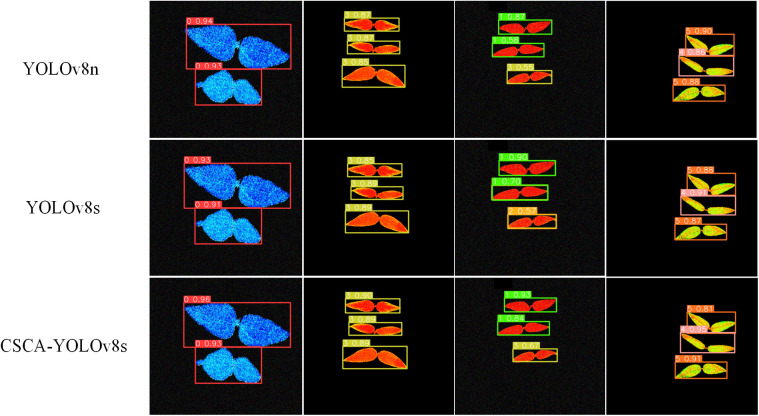
The actual detection results of CSCA-YOLOv8 compared with YOLOv8.

Fig 9 provides the confusion matrix plots of YOLOv8n, YOLOv8s, and CSCA-YOLOv8 models on different drought resistance level categories. The row represents the true drought resistance level category (True) and the column represents the predicted drought resistance level category (Predicted). The value of each element in the matrix represents the proportion of samples whose true drought level category is one class but predicted as another class. From the overall matrix, CSCA-YOLOv8s has a relatively higher proportion of correct classification in each category and fewer misjudgments. It shows that the model has better performance in identifying different drought resistance levels and can classify each class more accurately. In addition, in order to compare the detection results of different models more intuitively, we use the pre-trained weights to predict the recognition image. As shown in Fig 10, the red boxes represent the positions of the CK control group, and the green, yellow, orange, pink, and salmon-red boxes represent the positions where the model detects five types of drought resistance grade targets. Numbers represent the probability of detecting this type of drought resistance class. The higher the probability value, the more accurate the detection. It can be seen from Fig 10 that although YOLOv8n and YOLOv8s models perform very well in the actual process of drought resistance recognition, CSCA-YOLOv8s has the best overall performance in the actual target detection, and the confidence in each image is also high, so it can accurately identify the drought resistance of different mung bean varieties.

### Comparative experiments

To further evaluate the detection performance of the proposed method, we compare CSCA-YOLOv8 with the state of the art detection methods. It includes one-stage object detection models such as YOLOv5s, YOLOv9s, YOLOv10s, YOLOv11s, Efficientdet, and two-stage object detection models such as Faster RCNN. YOLOv5s is the smallest model in the YOLOv5 series, which has the characteristics of fast detection speed, lightweight and simple structure. YOLOv9s adopts advanced architecture design, including ELAN, PGI and other innovative technologies, which makes the model achieve remarkable results in the field of real-time object detection. YOLOv10 uses more convolutional layers and depthwise separable convolutions to improve model accuracy and computational efficiency. YOLOv10s can achieve fast inference speed with low hardware requirements by virtue of its fewer parameters and smaller model size. YOLOv11 is the latest version of the YOLO series. It not only achieves a comprehensive improvement in accuracy and speed, but also significantly enhances the model’s feature extraction capability by introducing the C2PSA and C3k2 modules. Compared with other versions, YOLOv11s can achieve faster inference speed and meet the needs of real-time detection. Faster RCNN is a two-stage object detection algorithm with high detection accuracy, but it has high computational complexity and slow detection speed. The EfficientDet model is an efficient object detection model proposed by the Google Brain team. The model uses EfficientNet as the backbone network and follows its compound scaling method to achieve an excellent balance between accuracy and speed in object detection tasks. The specific comparative experimental results are shown in Table 5.

**Table 5:** Performance comparison experiments with mainstream object detection algorithms.

Model	P (%)	R (%)	mAP@0.5 (%)	Layers	Parameter (M)	FLOPs (G)	FPS
Faster RCNN	78.68	92.02	92.27	225	82.37	28.57	1.59
EfficientDet	90.48	84.63	93.94	237	**3.83**	**5.21**	123.7
YOLOv5s	84.71	88.39	90.81	193	9.11	23.8	158.1
YOLOv9s	84.79	89.70	89.70	**486**	7.17	26.7	76.8
YOLOv10s	83.72	86.57	86.57	293	8.04	24.5	**165.2**
YOLOv11s	84.84	88.29	88.29	238	9.4	21.3	148.6
Ours	**91.05**	**92.17**	**94.75**	280	8.5	18.6	134.5

From Table 5, this paper first compares the model with Faster RCNN model and EfficientDet-D0 model. The experimental results show that the recall rate of the Faster RCNN model is as high as 92.02%, indicating that it has a strong ability to capture positive samples and is not easy to miss detection. However, the average accuracy of the model is only 78.68%, mAP@0.5 is 92.27%, and FPS is 1.59, which are 12.37%, 2.48%, and 132.91 lower than the CSCA-YOLOv8 model, respectively. In addition, the number of parameters of the Faster RCNN model is 82.37M, which is almost 10 times the volume of the CSCA-YOLOv8 model, which means that the model requires more calculation, has high hardware requirements, and is limited in practical application. On the contrary, the EfficientDet-D0 model has the smallest size of about 3.83M, which facilitates the deployment on resource-constrained devices. Although the precision and mAP@0.5 of the model have little difference within 1%, its recall value is 84.63%, which is 7.54% lower than that of the CSCA-YOLOv8 model, indicating that the model has weak ability to capture positive samples and may miss detection.

This paper also compares the modeling effects of typical models in the YOLO series. Compared with the CSCA-YOLOv8 model, the mAP@0.5 of the YOLOv5s model is reduced by 3.94%, and the number of parameters and FLOPs are increased by 0.61M and 5.2G, respectively. It shows that CSCA-YOLOv8 has higher accuracy and better control of parameter quantity and calculation amount in the detection task of mung bean drought resistance level. The accuracy and mAP@0.5 of YOLOv9s model are 84.79% and 89.70%, respectively, which are 6.26% and 5.05% lower than those of CSCA-YOLOv8 model. It shows that CSCA-YOLOv8 has obvious advantages in accuracy. Although the number of parameters is slightly more, the detection effect is significantly improved. Compared with YOLOv10s, the accuracy of CSCA-YOLOv8 model is improved by 7.33%, mAP@0.5 is improved by 8.18%. Although the CSCA-YOLOv8 model has shortcomings in the number of parameters and FPS, it is significantly ahead in the key detection accuracy indicators, which is more important for accurately detecting the drought resistance level. Although the YOLOv11s model is higher than CSCA-YOLOv8 in FPS, it is far inferior in other performance indicators.

Through the analysis, it can be concluded that the proposed detection method is superior to other target detection models in the detection task of mungbean drought resistance level. Compared with other advanced object detection models, the CSCA-YOLOv8 model not only shows a faster detection speed, but also achieves a higher average detection accuracy. The number of parameters of the model is 8.5M, and the frame rate (FPS) reaches 134.5. These advantages enable the CSCA-YOLOv8 model to be effectively used in complex field environments, which is beneficial to accurately detect the drought resistance level of mung bean varieties.

### Ablation experiments

#### Effect of different loss functions on the CSCA-YOLOv8 model.

To study the influence of different loss functions on the proposed method and determine the optimal loss function to optimize the model, this paper selects the CIoU, DIoU, EIoU, GIoU, PIoU and SIoU loss functions for comparative experiments. The specific comparative experimental results are shown in Table 6. From Table 6, when the loss function of CSCA-YOLOv8 is replaced from CIoU to EIoU and GIoU, the training time is slightly reduced. This is because the computational complexity of EIoU and GIoU is relatively reduced and the training iteration speed is accelerated in the process of optimizing bounding box regression. However, the accuracy of the model and mAP@0.5 both decreased slightly, indicating that although the training accelerated, the performance of the model detection accuracy became worse. This is because the modified constraints on bounding box regression reduced the model’s accuracy in predicting target locations. When PIoU is used, P (precision), R (recall), mAP@0.5 are all reduced, and the training time is not significantly improved. This indicates that the PIoU (Precise IoU) loss function fails to effectively optimize the model for this specific task. This demonstrates that the PIoU loss function fails to effectively optimize the model for mung bean drought resistance identification tasks. The SIoU loss function improves the performance of the model the most. Compared with DIoU, SIoU improves the accuracy of the model by 0.32%. The reason is that SIoU comprehensively considers scale invariance and other factors, which can make the model distinguish different drought resistance levels more accurately and reduce false detection and missed detection. The training time is reduced by 0.155h, because its optimization mechanism is more efficient and accelerates the convergence speed of the model. The FPS increases from 101.8 to 134.5, which indicates that SIoU is significantly effective in improving the detection speed. The model can process more image frames per second without reducing the very accuracy, which achieves an effective balance between the detection speed and the average accuracy. Therefore, the experimental results show that the SIoU loss function can accelerate the convergence of the model when the CSCA-YOLOv8 model is used for the mung bean drought resistance level detection task, and make the model reach a better performance state faster in the training process.

**Table 6:** Experimental results of various loss functions.

Model	P (%)	R (%)	mAP@0.5 (%)	t (hour)	FPS
CSCA-YOLOv8-CIoU	90.73	92.15	94.73	1.558	101.8
CSCA-YOLOv8-DIoU	90.17	**92.50**	**94.76**	1.494	126.1
CSCA-YOLOv8-EIoU	89.79	92.40	94.48	1.422	131.1
CSCA-YOLOv8-GIoU	89.65	92.03	94.39	1.404	126.0
CSCA-YOLOv8-PIoU	89.67	92.07	94.28	1.629	115.4
CSCA-YOLOv8-SIoU	**91.05**	92.17	94.75	**1.403**	**134.5**

#### Effect of C2f_Star_CAA instead of C2f_Star at different positions in the neck.

In order to study the influence of introducing C2f_Star_CAA modules at different positions on the CSCA-YOLOv8 model, we replace C2f_Star modules at four positions in the neck module with C2f_Star_CAA modules respectively, and conduct experiments on the MDD dataset. The results of the comparative experiments are shown in Table 7. It can be found from Table 7 that after replacing the C2f_Star module in position A with the C2f_Star_CAA module, the mAP@0.5 value of the model reaches the highest 94.85%, which indicates that the replacement of position A has certain advantages in measuring the comprehensive detection accuracy index of the model in different categories. However, its detection accuracy is only 90.38%, which is 0.67% lower than that of the model with D position replacement (91.05%). However, its detection accuracy is only 90.38%, which is 0.67% lower than that of the model with D position replacement (91.05%). This indicates that although the model after introducing the CAA module at position A has a prominent performance on mAP@0.5, it has shortcomings in the accuracy rate, which may lead to more false detection cases in the actual detection of the model. When the C2f_Star module at position B or C is replaced with the C2f_Star_CAA module, the mAP@0.5 values of the two models are within 0.1% of the model with the replacement at position D.It shows that in terms of comprehensive detection accuracy, the effect after replacing these two positions is similar to that after replacing D position. However, the training time increases by 0.514h and 0.478h respectively, which means that module replacement at these two positions will increase the model training time, because the computational complexity of the new module at this position increases.

**Table 7:** Comparison results of C2f_Star_CAA replacing C2f_Star in different positions.

Model					P (%)	R (%)	mAP@0.5 (%)	t (hour)	FLOPs (G)	FPS
Position A	√				90.38	**92.55**	**94.85**	1.439	18.6	121.5
Position B		√			90.73	**92.55**	94.73	1.917	18.7	117.4
Position C			√		90.51	92.04	94.66	1.881	18.6	119.6
Position D				√	**91.05**	92.17	94.75	**1.403**	18.6	**134.5**

By comprehensively comparing the model performance after replacing the C2f_Star module with the C2f_Star_CAA module at the four positions of A, B, C and D, the model after replacing the C2f_Star_CAA module at the position D showed the best overall detection performance in the mung bean drought resistance level detection task.

#### Performance analysis of different lightweight backbone networks.

Table 8 shows the experimental results of YOLOv8 model after adding different backbone networks. It can be seen from Table 8 that the model after adding the StarNet network performs well in precision (89.56%), recall (91.98%) and mAP@0.5 (94.37%). The number of parameters is 6545090, which is relatively small, and the FLOPs is 17.3G, which means the computational complexity is low. The FPS value is up to 195.4, and the detection speed is fast. Although the training time is slightly more than that of FasterNet, StarNet performs well in balancing model accuracy, computing resources and detection speed, which makes YOLOv8s run more efficiently and accurately in the detection of mung bean drought resistance level. The overall performance of the model introduced by the FasterNet network ranks second, but the detection accuracy is 0.4 percentage lower than that of YOLOv8s, which is because the lightweight design causes the model to lose some useful feature information. The recall values of EfficientViT and LSKNet are slightly higher than those of YOLOv8s, but the precision, mAP@50 and FPS values are generally lower than those of the baseline model, which indicates that not all backbone networks are suitable for YOLOv8s, and improper selection will affect the performance of the model. A comprehensive comparison of the performance of different backbone networks added to YOLOv8s shows that StarNet performs better in various key indicators, which can effectively control the number of parameters and computational complexity while ensuring the accuracy of model detection.

**Table 8:** Comparative results of different lightweight backbone networks.

Model	P (%)	R (%)	mAP@0.5 (%)	Parameter	t (hour)	FLOPs (G)	FPS
Baseline	88.53	90.72	93.62	11166560	1.943	28.8	132.3
YOLOv8s+EfficientViT	87.94	90.84	93.23	8383586	1.786	20.4	53.4
YOLOv8s+FasterNet	88.36	91.44	94.01	8618150	**1.128**	21.7	175.4
YOLOv8s+LSKNet	86.13	91.19	93.26	10306192	3.271	30.7	63.8
YOLOv8s+StarNet	**89.56**	**91.98**	**94.37**	**6545090**	1.188	**17.3**	**195.4**

#### Ablation study of the CSCA-YOLOv8 model.

**Fig 11:**
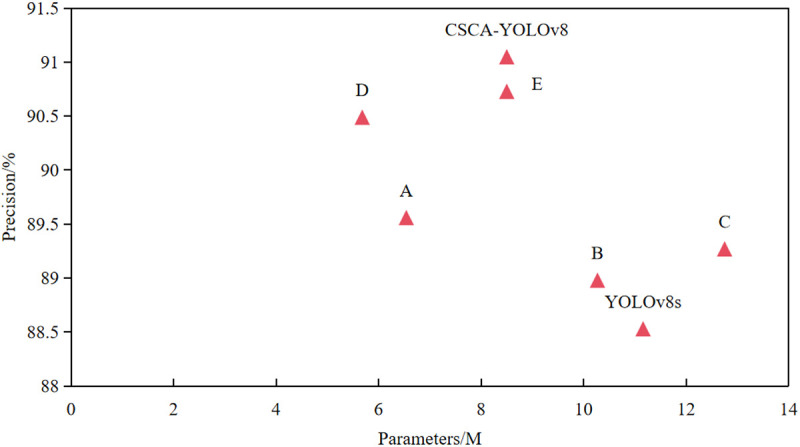
Scatter plot of precision and parameters for different improvement strategies.

In order to verify the credibility and effectiveness of YOLOv8s model after introducing StarNet, C2f_Star, CAA attention mechanism, CBGD module and SIoU loss function, we used YOLOv8s as the baseline model and carried out six groups of ablation experiments on the MDD dataset. The models are named A, B, C, D, E and F, respectively. The results of ablation experiments are shown in Table 9. Where√ represents that the module is used. In group A strategy, after introducing StarNet network into the backbone network of YOLOv8, the average accuracy and mAP@0.5 value of the model were increased by 1.03 and 0.75 percentage points respectively, and the parameters and FLOPs of the model were reduced by 41% and 40%, respectively. This shows that the StarNet network can minimize the number of network parameters and computational complexity while ensuring accuracy, making the model more lightweight and practical. In group B strategy, the C2f_Star module is used to reconstruct the original C2f module. Compared with the YOLOv8s model, the parameter amount and calculation amount of the model are reduced by 0.89M and 2.1G respectively, and the overall detection performance is also slightly improved. It shows that the C2f_Star module can improve the model accuracy and optimize the model structure to a certain extent while reducing the number of model parameters. Then, in group C, the CGBD module is integrated into the neck module of YOLOv8s. Although the number of parameters and calculation of the model are increased by 14% and 6%, the detection accuracy of the model is improved by 0.74 percentage points. It shows that although the CGBD module increases a certain amount of calculation and model scale, it can effectively improve the accuracy of model detection, which is due to the use of multi-scale structure to enhance the ability of model spatial feature extraction. In group D, the C2f_Star module is added on the basis of group A, and the accuracy is further improved to 90.49%, which is 0.93 percentage points higher than group A, and the accuracy is improved to 94.64%, which is 0.27 percentage points higher than group A. The number of parameters is reduced by 0.86M, and the calculation amount is reduced by 1.7 G. It shows that the combination of StarNet and the C2f_Star module integrates the features of different subspaces through element-level multiplication, which promotes the information interaction of different Spaces. It improves the spatial feature extraction ability of the model and further optimizes the performance of the model. Compared with the YOLOv8s model, after introducing the CAA attention mechanism, the mAP@0.5 value of the E model reaches 94.73%, an improvement of 1.11 percentage points. This is because the CAA attention mechanism uses global average pooling and bar convolution to enhance the features of the central region, so that the model can capture key information more accurately, thereby improving the detection accuracy. After adding SIoU loss function to group E, the accuracy and mAP@0.5 value of group F are improved again, reaching 91.05% and 94.75%, respectively. Combined with the previous experimental results, it can be seen that the SIoU loss function can further optimize the model to achieve a better balance between detection speed and accuracy. We also plot the scatter plots of the six strategies, as shown in Fig 11. Although the CSCA-YOLOv8 model has the highest average detection accuracy, the number of parameters still needs to be further optimized.

**Table 9:** The results of ablation experiments.

	StarNet	C2f_Star	CGBD	CAA	SIoU	P (%)	R (%)	mAP@0.5 (%)	Parameter (M)	FLOPs (G)	FPS
YOLOv8s						88.53	90.72	93.62	11.16	28.8	132.3
A	√					89.56	91.98	94.37	6.54	17.3	**195.4**
B		√				88.98	91.09	93.80	10.27	26.7	155.5
C			√			89.27	91.35	93.86	12.75	30.5	87.2
D	√	√				90.49	91.98	94.64	**5.68**	**15.6**	173.4
E	√	√	√	√		90.73	92.15	94.73	8.5	18.6	132.3
F	√	√	√	√	√	**91.05**	**92.17**	**94.75**	8.5	18.6	134.5

From the above analysis, it can be seen that the improved strategy has a positive impact on improving the performance of model detection, and the size of the model is also reduced. This highlights the effectiveness and authenticity of the proposed improved strategy in the drought resistance level recognition task of mung bean.

### Effects of PEG simulated stress on Fv/Fm parameters in mung bean resources

**Fig 12:**
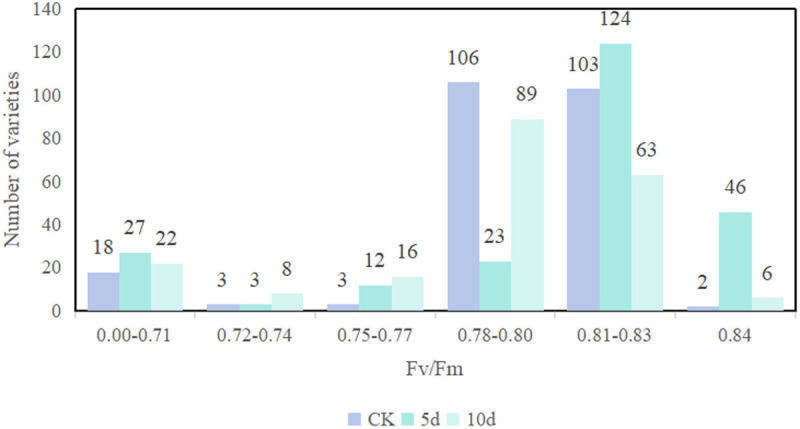
Quantitative distribution of mungbean varieties in different Fv/Fm intervals.

**Fig 13:**
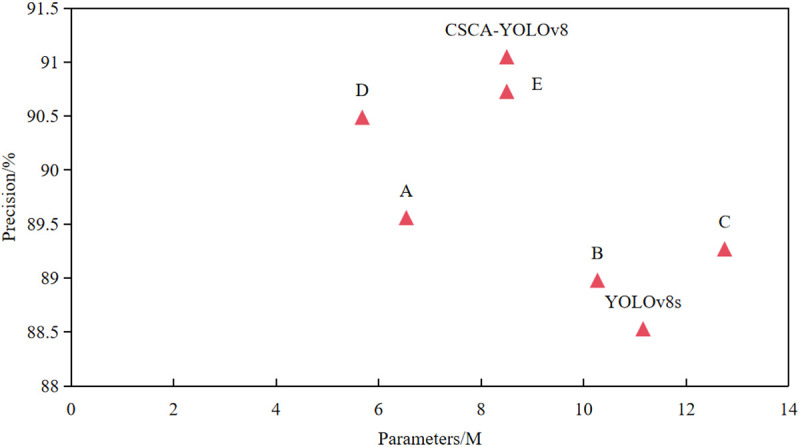
Changes in Fv/Fm of mungbean seedlings after drought stress simulated by PEG6000.

As shown in Fig 12, uunder normal physiological conditions, the Fv/Fm of mung bean varieties varies very little, usually between 0.78 and 0.83. Under stress conditions, the Fv/Fm ratio exhibits a characteristic biphasic response, initially increasing before subsequent decline. On the fifth day of drought stress, the Fv/Fm values of mung bean varieties generally ranged from 0.81 to 0.83. After the 10th day, the Fv/Fm of most mung bean varieties began to decrease in different ranges, basically between 0.78 and 0.8. Fig 13 shows the changing trend of Fv/Fm values of some mung bean seedlings with the extension of stress time. It can be seen from the figure that on the fifth day of stress treatment, the Fv/Fm of the four mung bean varieties all showed an upward trend. On the 10th day of drought stress, the Fv/Fm values of ‘Nenlü No.2’ and ‘Jilü No.7’ varieties decreased significantly, which was lower than that of CK group, indicating that the chloroplast structure was slightly damaged. However, the Fv/Fm values of ‘Zhonglu No.12’ and ‘Bao 865-18-9 74’ varieties decreased slightly, which indicated that these varieties were less affected by drought stress and had strong stability. PEG-simulated drought stress induces water deficit, leading to a reduction in Fv/Fm in mung bean, but with significant variation among cultivars.

### Screening of drought resistant germplasm resources of Mung bean

Superior drought-tolerant germplasm resources serve as the foundation for drought-resistant breeding and genetic research in mung bean. Based on 230 natural population materials of mung bean provided by Legumg Research Laboratory, Institute of Cash Crops, Jiangsu Academy of Agricultural Sciences, and according to the classification criteria of drought resistance, 9 cultivars with strong drought resistance and 31 cultivars with drought resistance after 5 days of drought stress were selected, accounting for 17.02% of the total. After ten days of drought stress, 7 extremely drought resistant varieties and 26 drought resistant varieties were screened out, accounting for 14.04% of the total. Among them, 4 varieties showed strong drought resistance at 5 and 10 days of drought stress, namely ‘Sulu No.6’, ‘LZL035 North2 D0797’, ‘Zhonglu No.12’ and ‘Bao 865-18-9 74’. In the future, we will enhance the collection of germplasm resources and identify drought-resistant varieties through rigorous drought resistance assessments. Concurrently, we must leverage the identified drought-resistant germplasm to intensify breeding efforts aimed at developing high-yielding mungbean varieties that are also drought-resistant.

## Conclusion

In order to realize the accurate detection of the drought resistance level of mung beans and meet the memory requirements of embedded devices, this study proposes a lightweight drought resistance identification method of mung beans based on chlorophyll fluorescence imaging technology and YOLOv8. The backbone network is replaced by StarNet and a new feature extraction module C2f_Star is constructed to replace C2f module, which not only reduces the number of parameters and computation, but also improves the detection ability of the model. The common convolution is reconstructed and optimized by using the feature fusion module CGBD. The local features and global features in the fluorescence image are extracted and fused respectively through the dual-branch structure. The robustness of the model is enhanced, and the detection accuracy of the model for the target is improved. In addition, we also introduce the CAA attention mechanism into the C2f_Star module, so that the model can focus on more feature information, reduce the interference of irrelevant information, and improve the target positioning ability and feature extraction ability of the model. The SIoU loss function is used to replace the CIoU loss function, which increases the model’s consideration of the scale and Angle information of the bounding box, and effectively accelerates the convergence speed and prediction accuracy of the model. The experimental results show that the CSCA-YOLOv8 model shows excellent detection performance on the self-built MDD dataset, and the parameter number and calculation amount are greatly reduced, which realizes the lightweight design of the model and provides the possibility for the method proposed in this study to be actually deployed on edge devices and embedded devices with limited computing power. Furthermore, this study also constructs a dataset of mungbean with 4808 fluorescence images. It need be pointed out that there is no publicly available drought stress fluorescence dataset so far. The MDD dataset can provide a reference for the subsequent research of genetic mapping of drought stress-related genes.

The method proposed in this study has certain limitations, which are primarily reflected in the following three aspects: (1)After integrating the CGBD module into the network, the model’s parameter count rises from 11.16 million to 12.75 million, while FLOPs experiences a boost from 28.8G to 30.5G. This is probably because the CGBD module adopts a two-branch structure, and each branch requires performing vertical and horizontal parameter calculations, thereby increasing the calculation cost. (2) The false detection of similar target categories still exists. (3) Due to the lack of public crop drought stress fluorescence datasets, the method proposed in this study can only verify its detection performance on self-built dataset, and it is difficult to verify its generalization performance on other datasets.

In the future, we will keep refining the network structure. A more efficient and lightweight CBGD module will be designed to improve the model’s capability in extracting features from fluorescence images under drought stress of mungbean. At the same time, we will continue to enrich the dataset to improve the generalization performance and robustness of the model. Furthermore, in order to enhance the generalization performance of the model, we will apply the method proposed in this paper to more complex scenarios.
